# Quality of Life in Adolescents with Schizophrenia: The Importance of Social Cognition and Residual Psychopathology in the Context of CYP2D6 Polymorphism

**DOI:** 10.3390/healthcare14111574

**Published:** 2026-06-04

**Authors:** Bianca Oana Bucatos, Nilima Rajpal Kundnani, Liana Dehelean, Ana-Maria Romosan, Radu Romosan, Adriana Cojocaru, Melania Veronica Ardelean, Maria Proks, Laura Alexandra Nussbaum

**Affiliations:** 1 Department of Neurosciences-Psychiatry, “Victor Babes” University of Medicine and Pharmacy, 300041 Timisoara, Romania; bianca.bucatos@umft.ro (B.O.B.); lianadeh@umft.ro (L.D.); romosan.ana@umft.ro (A.-M.R.); romosan.radu@umft.ro (R.R.); 2 PhD School Department, Victor Babes University of Medicine and Pharmacy, 300041 Timisoara, Romania; 3 University Clinic of Internal Medicine and Ambulatory Care, Prevention and Cardiovascular Recovery, Department VI—Cardiology, “Victor Babeș” University of Medicine and Pharmacy, 300041 Timisoara, Romania; 4 Research Centre of Timisoara, Institute of Cardiovascular Diseases, “Victor Babeș” University of Medicine and Pharmacy, 300041 Timisoara, Romania; 5 Centre for Cognitive Research in Neuropsychiatric Pathology, “Victor Babes” University of Medicine and Pharmacy, 300041 Timisoara, Romania; 6 Department of Neurosciences-Pedopsychiatry, “Victor Babes” University of Medicine and Pharmacy, 300041 Timisoara, Romania; adriana.cojocaru@umft.ro (A.C.); nussbaum.laura@umft.ro (L.A.N.); 7 1st Medical Semiology, Internal Medicine, Department V, Center for Advanced Research in Cardiovascular Pathology and in Hemostaseology, “Victor Babes” University of Medicine and Pharmacy, 300041 Timisoara, Romania; ardelean.melania@umft.ro; 8 Department of Pharmacology, “Victor Babes” University of Medicine and Pharmacy, 300041 Timisoara, Romania; proks.maria@umft.ro

**Keywords:** adolescent schizophrenia, quality of life, social cognition, theory of mind, CYP2D6 polymorphism, pharmacogenetics, negative symptoms

## Abstract

Background and Objectives: Schizophrenia in adolescence severely impairs quality of life (QoL) and disrupts critical social, academic, and neurodevelopmental periods. Beyond symptom remission, functional recovery and subjective well-being have become central therapeutic goals; however, the specific contributions of pharmacogenetic, social–cognitive, and residual clinical factors to QoL in early-onset schizophrenia remain largely unexplored. This study investigated the contribution of CYP2D6 metabolizer status, social cognition (theory of mind and empathy), residual psychopathology, and family factors to subjective QoL in adolescents with schizophrenia. Materials and Methods: A cross-sectional study of 52 adolescents with DSM-5 schizophrenia (PAT-SCZ) and 51 matched healthy controls. QoL was measured with the Pediatric Quality of Life Enjoyment and Satisfaction Questionnaire (PQ-LES-Q). Social cognition was assessed using the revised Reading the Mind in the Eyes Test (RMET). Residual symptoms were rated with the Positive and Negative Syndrome Scale (PANSS). CYP2D6 genotyping classified patients as normal metabolizers (NMs) or reduced-function metabolizers (RM: intermediate/poor). Two multivariable OLS regression models examined predictors of QoL. Results: Patients showed markedly lower QoL, impaired theory of mind, lower empathy, and higher residual symptoms than controls (all *p* < 0.0001). Within patients, reduced-function metabolizer (RM) status was associated with poorer QoL, weaker social cognition, higher negative/general psychopathology, and lower IQ. Two multivariable regression models explained 84.2–84.3% of the variance in QoL (adjusted R^2^). Social cognition (RMET scores) and CYP2D6 reduced-function metabolizer status emerged as the strongest predictors (β = 0.363 and β = −0.362, respectively, both *p* < 0.0001), followed by negative symptom severity and dysfunctional family relationships (all *p* < 0.05). Conclusions: Pharmacogenetic (CYP2D6), social–cognitive, and clinical factors strongly determine QoL in adolescent schizophrenia. Routine CYP2D6 genotyping, social-cognition remediation, and family interventions should be integrated into early treatment to improve long-term well-being.

## 1. Introduction

Schizophrenia is a severe, chronic psychiatric disorder characterized by disturbances in perception, thought, affect, cognition, and behavior, with onset most commonly occurring during late adolescence or early adulthood. Early-onset schizophrenia is associated with particularly unfavorable developmental trajectories, given that it disrupts critical periods of social, academic, and neurobiological maturation. Beyond symptom remission, contemporary treatment paradigms increasingly emphasize functional recovery and subjective quality of life (QoL) as central therapeutic outcomes. Indeed, symptom reduction alone does not fully capture patients’ lived experience, social integration, or perceived well-being.

Meta-analytic evidence demonstrates that psychiatric symptom severity shows only modest-to-moderate associations with subjective QoL in schizophrenia, indicating that QoL represents a multidimensional construct that cannot be reduced to symptom burden alone [1,2]. Eack and Newhill [1] found that while greater symptom severity is associated with poorer QoL, symptoms account for only a portion of the variance in patient-reported well-being. Similarly, Watson et al. [2], in a meta-analysis of first-episode psychosis, identified associations between total symptom severity, duration of untreated psychosis, and reduced QoL, yet substantial unexplained variance remained. These findings underscore the contribution of additional clinical, psychosocial, and biological determinants to overall well-being.

The relative contribution of specific symptom domains further refines this understanding. Negative symptoms and general psychopathology appear more strongly associated with reduced QoL than positive symptoms [1,2]. In first-episode schizophrenia and clinical high-risk populations, depressive and anxiety symptoms significantly predict lower QoL [3,4]. Long-term follow-up data indicate that individuals with adolescent-onset schizophrenia experience markedly impaired adult QoL compared to those with affective psychotic disorders [5]. Importantly, persistent negative symptoms, such as social withdrawal, diminished motivation, and affective flattening, are now recognized as major drivers of long-term disability and poor functional recovery in schizophrenia [6]. Negative symptom burden contributes substantially to impaired community functioning and subjective dissatisfaction, highlighting the need to consider residual psychopathology when evaluating QoL outcomes.

In parallel with symptom-based models, social cognition has emerged as a central determinant of functional outcome in schizophrenia. Social cognition encompasses theory of mind (ToM), emotion recognition, empathy, and attributional style—abilities that allow individuals to interpret social cues and navigate interpersonal contexts effectively. Increasing evidence supports the view that social cognition is a key driver of functional recovery in schizophrenia, often mediating the relationship between neurocognition and real-world functioning [7]. Javed and Charles emphasize that social cognition plays a pivotal role in overall recovery, with impairments strongly linked to poorer social functioning and community integration [7].

Meta-analytic findings confirm large effect-size deficits across multiple domains of social cognition in schizophrenia [8]. Importantly, social cognition predicts functional outcomes above and beyond neurocognition alone [9]. Fett et al. demonstrated that social–cognitive abilities show stronger associations with real-world functioning than traditional neurocognitive measures [9]. Furthermore, social cognition accounts for unique variance in everyday functioning [10], reinforcing its independent clinical relevance.

Beyond functional outcome, emerging evidence indicates that social cognition may directly influence subjective quality of life. In a large multi-site cohort study, Maat et al. [11] found that ToM performance significantly predicted subjective QoL in individuals with schizophrenia, whereas basic emotion recognition did not independently predict QoL. These findings suggest that higher-order mentalizing abilities may be particularly relevant for perceived well-being, linking interpersonal understanding directly to subjective life satisfaction.

These higher-order mentalizing difficulties appear early in the illness course. For instance, Ebisch et al. demonstrated altered neural mechanisms of social perception at a pre-reflective level in first-episode schizophrenia patients, characterized by disturbances in self-experience and impaired embodied simulation of others’ actions. Such findings underscore the relevance of social–cognitive deficits already present in the earliest stages of psychosis and provide a neurobiological backdrop for the behavioral impairments observed in our adolescent sample [12].

Empathy, another dimension of social cognition, is frequently impaired in schizophrenia. The Empathy Quotient (EQ) measures both cognitive and affective components of empathy [13,14], with cross-cultural validation confirming strong internal consistency [15]. Meta-analyses report medium-to-large empathy deficits in schizophrenia [16], although sex differences commonly observed in the general population may not consistently replicate in clinical samples [17].

While psychosocial determinants of QoL have been extensively studied, biological moderators remain comparatively underexplored. Cytochrome P450 2D6 (CYP2D6) is a highly polymorphic enzyme responsible for metabolizing several commonly prescribed antipsychotics, including risperidone and aripiprazole [18]. Genetic variation in CYP2D6 results in distinct metabolizer phenotypes (poor, intermediate, normal, ultrarapid), which influence plasma drug concentrations, therapeutic response, and adverse effect burden [18,19]. International pharmacogenetic guidelines recommend dose adjustments based on CYP2D6 genotype for certain psychotropic medications [19].

Emerging longitudinal evidence suggests that CYP2D6 metabolic activity may also influence long-term clinical trajectories. Sandhu et al. [20] reported that higher CYP2D6 activity was associated with more severe positive symptom trajectories and greater cognitive impairment over time in schizophrenia spectrum disorders. Individuals with high metabolic activity exhibited significantly greater odds of persistent cognitive deficits. These findings suggest that the CYP2D6 genotype may indirectly influence functional outcomes and subjective well-being through its effects on symptom persistence and cognition.

Given that cognitive and social–cognitive abilities are strong determinants of functional recovery and QoL [7,8,9,10,11,21], pharmacogenetic variation affecting cognitive or symptom trajectories may ultimately shape subjective well-being. Additionally, residual negative symptoms, known to drive poor functional outcomes [6], may further mediate the relationship between biological vulnerability and QoL. Despite these plausible mechanistic pathways, few studies have integrated psychosocial and pharmacogenetic predictors of QoL, particularly in adolescent populations.

Importantly, most prior research examining QoL, social cognition, and pharmacogenetics has focused on adult samples. Adolescent-specific data integrating residual psychopathology, social–cognitive functioning, family context, and CYP2D6 metabolizer status remain scarce. Given the developmental sensitivity of adolescence and the profound long-term implications of early-onset schizophrenia, understanding the interplay between biological and psychosocial determinants of QoL in this population is of particular clinical relevance.

### Objectives

The present study, therefore, aims to investigate the quality of life in adolescent patients diagnosed with schizophrenia who were genetically tested for metabolic abnormalities related to CYP2D6 mutations (normal metabolizers versus intermediate/poor metabolizers). Another objective was to study psychosocial (mentalizing abilities, also known as ToM, empathy levels, family relationships, family history of psychiatric disorders) and clinical (residual psychopathology) factors that could significantly predict quality of life in these patients. By integrating pharmacogenetic and psychosocial dimensions, this study seeks to clarify the pathways through which biological and clinical factors converge to influence quality of life in adolescent schizophrenia.

## 2. Materials and Methods

This cross-sectional study included 52 adolescents diagnosed with schizophrenia (PAT-SCZ group) and 51 healthy controls (HC group) matched for age, sex, and intelligence quotient (IQ). Exclusion criteria were refusal to participate and active substance abuse. Diagnoses were established according to DSM-5 criteria by experienced child and adolescent psychiatrists. All participants and their legal guardians provided written informed consent. The study protocol was approved by the local institutional ethics committee and conducted in accordance with the Declaration of Helsinki.

The following data were collected for each participant: gender, age, metabolizer status (metabolic abnormalities related to CYP2D6 mutations: normal metabolizers versus intermediate/poor metabolizers), family history of psychiatric disorders (psychiatric conditions such as schizophrenia, schizoaffective disorder, bipolar disorder, recurrent depressive disorder, and substance abuse), psychosocial context (intrafamilial relationships—dysfunctional families versus stable families).

Psychosocial context was qualitatively assessed during the psychiatric interview with regard to intrafamilial relationships. These were classified as either stable family relationships (characterized by supportive, cohesive interactions and adequate emotional support) or dysfunctional family relationships (characterized by persistent conflict, lack of support, fragmented communication, or high expressed emotion). This binary classification was dichotomized (0 = stable, 1 = dysfunctional) for inclusion in the regression analyses.

Quality of life was assessed using the Pediatric Quality of Life Enjoyment and Satisfaction Questionnaire (PQ-LES-Q), a validated self-report instrument demonstrating high internal consistency (α ≈ 0.87–0.90) and good concurrent validity in youth populations [22]. The PQ-LES-Q is derived from the original Quality of Life Enjoyment and Satisfaction Questionnaire (Q-LES-Q) [23], a widely used instrument assessing subjective satisfaction across multiple life domains. Reliability of Q-LES-Q-based measures has been confirmed in schizophrenia samples [24], and abbreviated forms such as the Q-LES-Q-18 exhibit strong psychometric properties [25].

Residual psychopathology was evaluated using the Positive and Negative Syndrome Scale (PANSS) [26]. The PANSS comprises 30 items divided into positive, negative, and general psychopathology subscales and remains the gold standard for symptom assessment in schizophrenia. Its applicability to pediatric populations is supported by validation of the Kiddie-PANSS [27], and more recent work confirms the reliability and treatment sensitivity of abbreviated PANSS forms in pediatric psychopharmacology trials [28].

Theory of mind abilities were assessed using the revised Reading the Mind in the Eyes Test (RMET) [29]. The RMET has demonstrated validity in distinguishing clinical populations [29], robust psychometric properties [30], and consistent performance deficits in schizophrenia across meta-analytic studies [31]. Normative age- and sex-specific data facilitate developmental interpretation in adolescent samples [32]. Empathy levels were evaluated with the Cambridge Empathy Quotient (EQ), an established self-administered questionnaire that gauges both cognitive and affective empathy [13]. Cognitive intelligence was measured employing Raven’s Progressive Matrices, a reliable instrument for assessing nonverbal reasoning skills [33].

Genomic DNA was extracted from peripheral blood samples using validated molecular procedures. CYP2D6 genotyping was performed using established laboratory techniques. Participants were classified according to predicted metabolizer phenotype following international consensus activity score recommendations [34] and Dutch Pharmacogenetics Working Group guidance [19]. Antipsychotic treatment was recorded and categorized based on CYP2D6 metabolic involvement and pharmacogenetic relevance [18,19].

### Statistical Analysis

Data were analyzed using IBM SPSS Statistics (version 20; IBM Corporation, Armonk, NY, USA). As the normality tests (Shapiro–Wilk and Kolmogorov–Smirnov) indicated mostly a non-Gaussian data distribution (*p* < 0.05), differences between groups were examined using non-parametric tests (Mann–Whitney U test). Spearman’s correlation analysis was used to seek correlations between scale scores, while the χ^2^ test was performed to assess categorical variables.

Multivariable linear regression analyses were performed to identify predictors of quality of life (PQ-LES-Q). Model fit was assessed via the F-statistic and Adjusted R^2^. Individual predictors were evaluated using unstandardized (B) and standardized (β) coefficients. Assumptions were verified using the Durbin-Watson statistic for residual independence and variance inflation factors (VIFs) for multicollinearity. To ensure the robustness of the estimates given the sample size, internal validation was performed using 1000 non-parametric bootstrap resamples to generate Bias-Corrected and accelerated (BCa) 95% confidence intervals. Two models were compared: model 1 utilized the PANSS total score, while model 2 utilized the three PANSS subscales. Pharmacogenetic profile (metabolizer status) was treated as a binary variable, where 0 = Normal/Extensive Metabolizers and 1 = Poor/Intermediate Metabolizers (allowing the regression coefficient to represent the specific impact of genetic metabolic variation on quality of life). The psychosocial context was qualitatively assessed during the psychiatric interview regarding intrafamilial relationships. These descriptions were dichotomized for inclusion in the regression analysis, categorized as either ‘stable’ (characterized by supportive, cohesive interactions) or ‘dysfunctional’ (characterized by persistent conflict, lack of support, or fragmented communication). This binary classification allowed for the assessment of the family environment’s discrete impact on the patient’s reported QoL (0 = stable family relationships and 1 = dysfunctional family relationships). RMET was utilized as a continuous measure of ToM, with higher total scores indicating superior mental state recognition. PANSS was utilized both as a global total score (Model 1) and via its three validated subscales (Model 2): Positive (P), Negative (N), and General Psychopathology (G).

A post hoc power analysis for multiple linear regression, based on the observed adjusted R^2^ values of approximately 0.843 (corresponding to a very large effect size, f^2^ ≈ 5.37), demonstrated that the sample size of N = 52 provided excellent statistical power (approaching 1.00) to detect the large effects found in both models at α = 0.05, even with up to 6 predictors.

For all statistical analyses, the level of significance was set at 0.05, with all results being two-tailed.

## 3. Results

The results for data normality tests are presented in Table 1 (Kolmogorov–Smirnov and Shapiro–Wilk).

The study included two main samples, the PAT-SCZ sample (52 adolescent patients diagnosed with schizophrenia) and the HC sample (51 healthy adolescents, matched for gender, age, and IQ).

Clinical and demographic data, as well as the results of the psychiatric assessment for PAT-SCZ and HC, are presented in Table 2, comparatively.

As revealed by Table 2, the PAT-SCZ showed significantly lower scores than the HC group in all the psychiatric assessment scales. They reported a significantly reduced quality of life, impaired mentalizing abilities, and lower empathy levels than their healthy counterparts.

### 3.1. Factors Impacting Quality of Life in Adolescent Patients with SCZ

Patients from the PAT-SCZ sample received the following antipsychotic treatment: 13 (25%) received aripiprazole, 11 (21.2%) received risperidone, 24 (46.2%) received olanzapine, and 4 (7.6%) received quetiapine. To simplify, patients were grouped into two categories as regards the effect of their antipsychotic medication on CYP2D6: 28 (53.8%) were on antipsychotics with low/minimal effect on CYP2D6 (olanzapine or quetiapine), while 24 (46.2%) received antipsychotics with moderate/high impact on CYP2D6 (aripiprazole or risperidone).

According to the genetic testing, the PAT-SCZ sample included 19 normal metabolizers (NMs), 31 intermediate metabolizers (IMs), and 2 poor metabolizers (PMs). Because the genetic analysis indicated only 2 PM, PAT-SCZ patients were classified as NM and metabolizers with reduced function (RM), which included both IM and PM patients.

In the PAT-SCZ sample, PQ-LES-Q scores were significantly higher in female patients (U = 152, Z = −3.34, *p* = 0.001). PQ-LES-Q scores were significantly lower in RM patients (U = 25, Z = −5.49, *p* < 0.0001), in patients treated with antipsychotics with moderate/high impact on CYP2D6 (U = 201, Z = −2.48, *p* = 0.01), in patients having a positive family history of psychiatric disorders (U = 213, Z = −2.28, *p* = 0.02) and in patients revealing dysfunctional family relationships (U = 150, Z = −3.28, *p* = 0.001). PQ-LES-Q scores correlated positively with RMET scores (Spearman’s rho [r] = 0.81, *p* < 0.0001), EQ-C scores (Spearman’s rho [r] = 0.53, *p* < 0.0001), EQ-AF scores (Spearman’s rho [r] = 0.59, *p* < 0.0001), EQ-SS scores (Spearman’s rho [r] = 0.61, *p* < 0.0001), EQ total scores (Spearman’s rho [r] = 0.63, *p* < 0.0001) and IQ (Spearman’s rho [r] = 0.62, *p* < 0.0001). PQ-LES-Q scores correlated negatively with PANSS-N scores (Spearman’s rho [r] = −0.73, *p* < 0.0001), PANSS-G scores (Spearman’s rho [r] = −0.71, *p* < 0.0001) and PANSS total scores (Spearman’s rho [r] = −0.81, *p* < 0.0001).

In order to do a more comprehensive assessment of the quality of life in the PAT-SCZ sample, it was further analyzed, divided into the two sub-samples according to metabolizer status: PAT-SCZ-NM (normal metabolizers) and PAT-SCZ-RM (reduced metabolizers).

We found no significant differences between PAT-SCZ-NM and PAT-SCZ-RM regarding gender, age, and choice of antipsychotic medication (low/minimal effect on CYP2D6 vs. moderate/high effect on CYP2D6). However, patients from the PAT-SCZ-RM sub-sample had a positive family history of psychiatric disorders (schizophrenia, bipolar or major depressive disorder, personality disorders) significantly more frequently than patients from the PAT-SCZ-NM group (χ^2^ = 8.79, *p* = 0.003). They also reported significantly more dysfunctional family relationships than the PAT-SCZ-NM patients (χ^2^ = 4.65, *p* = 0.03).

Psychiatric assessment results are provided in Table 3, comparatively for the PAT-SCZ-NM patients and the PAT-SCZ-RM patients.

In the PAT-SCZ-NM sub-sample, females obtained significantly higher PQ-LES-Q scores (U = 18, Z = −2.05, *p* = 0.04), RMET scores (U = 7.5, Z = −2.95, *p* = 0.003), EQ-C scores (U = 18.0, Z = −2.06, *p* = 0.04) and EQ total scores (U = 13.0, Z = −2.04, *p* = 0.01), while PANSS-N (U = 11.0, Z = −2.70, *p* = 0.007), PANSS-G (U = 12.0, Z = −2.62, *p* = 0.009) and PANSS total scores (U = 9.5, Z = −2.78, *p* = 0.006) were significantly lower than those of their male counterparts. PQ-LES-Q scores correlated negatively with age (Spearman’s rho [r] = −0.66, *p* = 0.002) and PANSS-G scores (Spearman’s rho [r] = −0.56, *p* = 0.01). On the other hand, PQ-LES-Q scores correlated positively with RMET scores (Spearman’s rho [r] = 0.68, *p* = 0.001), EQ-C scores (Spearman’s rho [r] = 0.49, *p* = 0.01), EQ-AF (Spearman’s rho [r] = 0.62, *p* = 0.005), EQ-SS (Spearman’s rho [r] = 0.54, *p* = 0.02), EQ total (Spearman’s rho [r] = 0.63, *p* = 0.004) and IQ scores (Spearman’s rho [r] = 0.59, *p* = 0.008). Family history of psychiatric disorders, family relationships, and antipsychotic treatment had no significant influence on PQ-LES-Q scores in the PAT-SCZ-NM sub-sample.

In the PAT-SCZ-RM sub-sample, PQ-LES-Q scores were significantly higher in patients receiving antipsychotics with low/minimal impact on CYP2D6 (U = 40.5, Z = −3.45, *p* = 0.001) than in patients treated with antipsychotics with moderate/high impact on CYP2D6. Moreover, RMET scores (U = 23.5, Z = −4.06, *p* < 0.0001), EQ-C scores (U = 1.00, Z = −4.89, *p* < 0.0001), EQ-AF scores (U = 42.0, Z = −3.44, *p* = 0.001), EQ-SS (U = 14.0, Z = −4.45, *p* < 0.0001) and EQ total scores (U = 1.00, Z = −4.87, *p* < 0.0001) were also significantly higher in patients that received antipsychotics with low/minimal impact on CYP2D6, while PANSS-G scores (U = 43.5, Z = −3.35, *p* = 0.001) and PANSS total scores (U = 51.5, Z = −3.05, *p* = 0.002) were significantly lower in these patients. PQ-LES-Q scores were significantly lower in patients reporting dysfunctional family relationships (U = 69.5, Z = −2.40, *p* = 0.02). PQ-LES-Q scores correlated positively with RMET scores (Spearman’s rho [r] = 0.76, *p* < 0.0001), EQ-C scores (Spearman’s rho [r] = 0.63, *p* < 0.0001), EQ-AF (Spearman’s rho [r] = 0.24, *p* = 0.02), EQ-SS (Spearman’s rho [r] = 0.62, *p* < 0.0001), EQ total (Spearman’s rho [r] = 0.63, *p* < 0.0001), IQ (Spearman’s rho [r] = 0.59, *p* < 0.0001) and negatively with PANSS-N (Spearman’s rho [r] = −0.47, *p* = 0.006), PANSS-G (Spearman’s rho [r] = −0.50, *p* = 0.003) and PANSS total (Spearman’s rho [r] = −0.65, *p* < 0.0001). PQ-LES-Q scores were not significantly influenced by age, gender, or family history of psychiatric disorders in the PAT-SCZ-RM sub-sample.

### 3.2. The Influence of Social Cognition, Residual Psychopathology, and Metabolizer Status on Quality of Life in Adolescent Patients with SCZ

To provide a comprehensive assessment of the predictors of quality of life, we utilized two distinct multivariable OLS regression models. To maintain model parsimony and ensure sufficient statistical power given the sample size (N = 52), we employed a targeted selection of predictors. Model 1 focused on four primary biopsychosocial domains and included global symptom severity (PANSS total scores) alongside biological (metabolizer status), socio-cognitive (RMET), and environmental (psychosocial context) predictors. Model 2 replaced the PANSS total score with its three constituent subscales (positive, negative, and general psychopathology) to identify the specific symptomatic dimensions that were most influential to subjective well-being. In Model 2, while the number of predictors was increased to six to allow for symptomatic granularity, the participant-to-variable ratio (approximately 9:1) remained within acceptable limits for exploratory clinical research. The stability of these models was further verified through multicollinearity diagnostics and bootstrap resampling.

The first multivariable OLS regression was conducted to identify biological, cognitive, and clinical predictors of QoL (PQ-LES-Q total). The model was highly significant, F(4, 47) = 68.83, *p* < 0.0001, explaining 84.2% of the adjusted variance in QoL (adjusted R^2^ = 0.842). The model met all standard assumptions for linear regression: the Durbin-Watson statistic of 1.96 indicates no significant autocorrelation, variance inflation factors (VIFs) for all predictors were between 1.23 and 2.54 (well below the conservative threshold of 3), while the participant-to-variable ratio of 13:1 ensured a stable and parsimonious model. All four predictors in the model reached statistical significance. Social cognition (RMET scores) was the strongest positive predictor (β = 0.363, *p* < 0.0001), higher RMET scores being associated with significantly better quality of life. Metabolizer status showed an almost equal, but inverse, strength of effect (β = −0.362, *p* < 0.0001), indicating that specific metabolizer profiles significantly diminish perceived quality of life. Global symptom severity (PANSS total) was also a significant negative predictor (β = −0.263, *p* = 0.005). However, its weight in the model was lower than that of RMET and metabolizer status. Psychosocial context (dysfunctional family relationships) also significantly impacted quality of life (β = −0.139, *p* = 0.028).

To validate the robustness of the primary model, we performed an internal validation using 1000 bootstrap resamples. The bootstrap results for the model summary yielded a narrow 95% confidence interval for the Durbin-Watson statistic and minimal bias. The bias-corrected accelerated (BCa) 95% confidence intervals confirmed the significance of all primary predictors. Notably, RMET scores (BCa 95% CI [0.276, 0.731]) and metabolizer status (BCa 95% CI [−11.464, −4.210]) demonstrated high stability with negligible bias. PANSS total scores (BCa 95% CI [−0.821, −0.039]) and psychosocial context (BCa 95% CI [−5.667, −0.026]) also remained significant predictors, as their respective confidence intervals did not cross zero. These results demonstrate that the high explanatory power of the model (adjusted R^2^ = 0.84) is stable across resampled distributions and is not an artifact of specific outliers within our cohort (Table 4).

When PANSS was decomposed into its constituent subscales (Model 2-Table 5), the model accounted for 84.3% of the adjusted variance and was statistically significant: adjusted R^2^ = 0.843, F(6, 45) = 46.78, *p* < 0.0001. Negative symptoms emerged as a significant negative predictor of quality of life (β = −0.20, *p* = 0.012), while positive (*p* = 0.40) and general symptoms (*p* = 0.08) did not reach statistical significance. Internal validation via 1000 bootstrap resamples confirmed that the symptomatic influence was localized specifically to negative symptoms (BCa 95% CI [−1.34, −0.07]). More importantly, the effects of social cognition (RMET scores) and metabolizer status remained robust and virtually unchanged from the global model, underscoring their status as primary, independent determinants of patient QoL. This stability suggests that social cognition and pharmacogenetic profile influence quality of Life through pathways that are independent of primary psychotic symptomatology.

A side-by-side comparison table integrating the standard coefficients and the bootstrap confidence intervals for the two proposed models is presented in Table 6.

## 4. Discussions

This cross-sectional study is among the first to integrate pharmacogenetic data (CYP2D6 metabolizer status), residual symptomatology, and psychosocial variables, including ToM and family functioning, when examining subjective QoL in adolescents diagnosed with schizophrenia. As anticipated, patients reported markedly lower quality of life, impaired ToM abilities, reduced empathy, and higher levels of residual symptoms compared with age-, sex-, and IQ-matched healthy controls (Table 2). Within the patient group, those classified as reduced-function metabolizers (intermediate and poor combined) exhibited substantially poorer QoL, weaker social–cognitive performance, more severe negative and general psychopathology, and lower IQ than normal metabolizers (NMs) (Table 3). The two multivariable regression models accounted for a large proportion of variance in PQ-LES-Q scores (adjusted R^2^ = 0.842–0.843), with social cognition (RMET total score) and CYP2D6 reduced-function metabolizer status emerging as the strongest and nearly equivalent independent predictors, followed by negative symptom severity and dysfunctional family relationships.

The prominent role of ToM in predicting QoL extends previous findings from adult populations to the critical developmental window of adolescence. While meta-analyses have long established large deficits in social cognition in schizophrenia and demonstrated its contribution to functional outcomes beyond neurocognition, direct associations with subjective well-being have been less frequently examined. Our results show that the ability to accurately infer others’ mental states (as measured by the RMET) remains a robust predictor even after controlling for negative symptoms, global psychopathology, and pharmacogenetic status. This finding aligns with evidence that higher-order mentalizing capacities are particularly relevant for perceived life satisfaction, especially during adolescence when peer relationships, school reintegration, and identity formation are central developmental tasks. The fact that empathy subscales, although correlated at the bivariate level, lost independent significance in the multivariable models suggests that the cognitive component of social cognition may be more proximal to subjective well-being than affective empathy in this age group. These observations reinforce the importance of early assessment and targeted remediation of social–cognitive deficits in early-onset psychosis.

The strong negative association between reduced CYP2D6 function and quality of life is consistent with growing evidence on how this genotype influences antipsychotic pharmacokinetics and tolerability in young people. Systematic reviews have shown that poor or intermediate metabolizers reach higher plasma concentrations of CYP2D6 substrate antipsychotics, experience more adverse reactions, and show poorer tolerability, especially with risperidone [35,36]. In our cohort, this disadvantage was particularly evident among patients prescribed moderate- to high-impact CYP2D6 substrates, whereas those receiving agents with minimal CYP2D6 involvement (olanzapine or quetiapine) showed better QoL scores, superior social–cognitive performance, and milder general psychopathology. A prospective study of 56 adolescents with psychosis reported the same pattern: normal metabolizers achieved significantly larger PANSS reductions over 12 months and had fewer adverse reactions than intermediate metabolizers [37]. These patterns support growing calls for pharmacogenetic-guided prescribing in adolescent psychiatry and are in line with prospective data showing better symptom reduction and fewer adverse reactions in normal metabolizers [18,19]. Nevertheless, the small number of poor metabolizers and the absence of plasma level monitoring and systematic side-effect assessment necessitate cautious interpretation.

The strong correlation between ToM performance (RMET) and QoL (r = 0.81; independent β = 0.27–0.31) is a new contribution. Meta-analyses have established large ToM deficits in schizophrenia and shown that social cognition predicts functional outcome beyond neurocognition alone [8,9,10]. Direct links to subjective well-being have been demonstrated mainly in adults, where higher-order mentalizing (but not basic emotion recognition) predicts life satisfaction [11]. Our data extend this pattern to adolescents, suggesting that the ability to infer others’ intentions and beliefs is especially relevant for perceived well-being at this age, when peer relationships, school reintegration, and identity formation are central. Empathy subscales correlated with QoL but lost significance once theory of mind and other variables were entered, indicating that the cognitive component of social cognition may be more proximal to subjective well-being in this developmental stage. The findings therefore strengthen the case for routine social–cognitive assessment and targeted remediation in early-onset psychosis.

Negative symptoms emerged as the only PANSS subscale that retained independent predictive value for poorer quality of life in the domain-specific model. This finding corroborates extensive literature indicating that avolition, social withdrawal, anhedonia, and diminished emotional expression constitute the primary drivers of long-term disability and reduced life satisfaction in schizophrenia, often exerting a stronger influence than positive symptoms once the latter are adequately controlled [6]. In adolescent and early-onset populations, negative symptoms are both highly prevalent and particularly detrimental to developmental trajectories, underscoring the need for interventions that go beyond dopamine D2 receptor blockade and specifically target motivational and expressive deficits [38].

Dysfunctional family relationships also exerted a significant independent negative effect on QoL, an influence that was more pronounced among reduced-function metabolizers, who, in turn, more frequently reported a positive family history of psychiatric disorders. This interplay highlights the biopsychosocial complexity of early-onset schizophrenia, whereby genetic vulnerability and adverse family context may amplify each other through mechanisms such as increased stress, reduced emotional support, and poorer treatment adherence. Our results are consistent with studies demonstrating that family environment and expressed emotion significantly shape functional and subjective outcomes in adolescent psychotic disorders [39].

An interesting secondary observation was the presence of sex differences favoring females within the normal-metabolizer subgroup, which manifested as higher QoL scores, better ToM performance, and milder negative and general symptoms. Although sex differences in schizophrenia outcomes are not always consistent across studies, these findings suggest possible protective effects of female sex on social cognition and symptom expression during adolescence that deserve further exploration in larger samples.

Taken together, the present findings support a multidimensional, precision-medicine framework for adolescent schizophrenia. Routine pre-treatment CYP2D6 genotyping could assist clinicians in selecting better-tolerated antipsychotics, thereby improving adherence and subjective well-being [35,37]. Concurrently, early identification of theory-of-mind difficulties should prompt specific social–cognitive remediation programs, many of which have shown promising results in improving social functioning. Family interventions aimed at enhancing communication, reducing conflict, and strengthening support networks appear equally important, particularly in families with elevated genetic loading. By addressing these interconnected domains, treatment strategies may move beyond symptom reduction toward genuine improvements in long-term QoL and functional recovery [39].

### Strengths and Limitations

Strengths of the study include the integration of pharmacogenetic, clinical, and psychosocial variables in a rarely studied adolescent population, the use of well-validated instruments, the inclusion of matched healthy controls, and statistical modeling that met all diagnostic assumptions. Although the sample is modest, the participants are well characterized and representative of clinical early-onset schizophrenia.

While these results are preliminary, the significant association between CYP2D6 metabolizer status and QoL scores underscores the potential importance of pharmacogenetic variation in psychiatric outcomes. This supports a hypothesis-generating framework where ‘side-effect burden’, potentially driven by metabolic rate, acts as a silent mediator of life satisfaction, independent of clinical symptom control. Our findings indicate that social cognition, specifically the ability to infer mental states (RMET), is a primary determinant of subjective well-being that may outweigh the impact of overt psychotic symptoms. This suggests that a patient’s capacity to navigate social nuances is more central to their perceived quality of life than the presence or absence of psychotic symptoms. By deconstructing the PANSS into its constituent subscales, we observed that the negative impact of symptoms was localized exclusively to the negative syndrome. This aligns with recent literature suggesting that ‘deficit’ symptoms (e.g., apathy, social withdrawal) create a more substantial barrier to functional recovery and life satisfaction than positive symptoms (6), which showed no significant predictive value in our bootstrapped models. Several limitations must be acknowledged. First, the cross-sectional design of this study precludes any definitive causal inferences, offering no insight into how these relationships may evolve over the course of the illness. Moreover, the modest sample size (N = 52), while statistically robust according to our internal bootstrap validation, necessitates that these findings be treated as preliminary evidence for the integration of social–cognitive and genetic markers in clinical practice. The remarkably high adjusted R^2^ values (0.842–0.843) raise legitimate concerns about potential overfitting and limited generalizability. In psychiatric research, models rarely explain more than 40–60% of the variance in QoL outcomes; our results should therefore be interpreted as exploratory and hypothesis-generating rather than definitive. While the significant impact of metabolizer status on QoL is compelling, the presence of residual confounding (e.g., lifestyle factors or polypharmacy) suggests that routine genotyping should be investigated further in larger, longitudinal cohorts before widespread clinical implementation is recommended. The classification of family relationships as “stable” versus “dysfunctional” relied on a clinically derived binary judgment during psychiatric interview rather than a validated structured instrument. This approach, while ecologically valid in a real-world clinical context, introduces potential observer bias and reduces reproducibility. We also lacked detailed data on duration of illness, duration of untreated psychosis, cumulative antipsychotic exposure (in chlorpromazine equivalents), exact dosages, side-effect burden, plasma concentrations, and socioeconomic status, variables that could confound both QoL ratings and metabolizer status comparisons. Although IQ was used for matching and showed strong bivariate correlations with QoL, it was not entered as a covariate in the final models to preserve degrees of freedom; sensitivity analyses (available upon request) indicated that its inclusion did not materially alter the primary findings.

CYP2D6 analyses grouped intermediate and poor metabolizers because only two participants were poor metabolizers. The absence of plasma concentration data and systematic side effect ratings further tempers the strength of conclusions regarding pharmacogenetic guidance. Finally, while sex differences emerged in the normal-metabolizer subgroup, the study was not powered to examine sex-by-metabolizer interactions formally. Future research should prioritize longitudinal designs, larger samples, comprehensive confounder assessment, and validated family-functioning measures to confirm and extend the present observations. We also acknowledge that our participant-to-variable ratio is at the lower end of traditional recommendations. However, the consistent significance of our primary predictors (RMET and metabolizer status) across both the four-variable and six-variable models, combined with robust results from 1000 bootstrap resamples, indicates that the findings are stable and not merely an artifact of the model’s complexity.

## 5. Conclusions

In conclusion, this study demonstrates that subjective quality of life in adolescents with schizophrenia is determined by an interplay of pharmacogenetic, clinical, and psychosocial factors. Social cognition (RMET) and reduced CYP2D6 metabolic function were the strongest predictors of poorer QoL, followed by negative symptom severity and dysfunctional family relationships. The regression models explained nearly 84% of the variance in PQ-LES-Q scores, confirming that symptom reduction alone is insufficient for meaningful recovery. These findings extend the largely adult literature to the adolescent period and provide one of the first multidimensional views of QoL in early-onset schizophrenia.

The results support a precision-medicine approach in this population. Routine pre-treatment CYP2D6 genotyping should guide antipsychotic selection and dosing to improve tolerability and well-being. Early assessment of theory of mind deficits and family difficulties should prompt targeted social–cognitive remediation and family interventions. Larger, multicenter, longitudinal studies are now needed to confirm these relationships over time and to evaluate whether combined genotype-guided treatment, social–cognitive training, and family support can enhance long-term functional recovery and quality of life.

## Figures and Tables

**Table 1:** Tests of normality (Kolmogorov–Smirnov and Shapiro–Wilk) for study variables in adolescents with schizophrenia.

Tests of Normality						
	Kolmogorov–Smirnov ^a^			Shapiro–Wilk		
	Statistic	df	Sig.	Statistic	df	Sig.
Age	0.233	52	0.000	0.836	52	0.000 **
RMET total score	0.174	52	0.000	0.894	52	0.000 **
EQ-C	0.091	52	0.200 *	0.953	52	0.039 *
EQ-AF	0.219	52	0.000	0.903	52	0.000 **
EQ-SS	0.117	52	0.072	0.956	52	0.052
EQ TOTAL	0.076	52	0.200 *	0.965	52	0.129
IQ	0.089	52	0.200 *	0.966	52	0.142
PANSS (P)	0.368	52	0.000	0.702	52	0.000 **
PANSS (N)	0.169	52	0.001	0.920	52	0.002 *
PANSS (G)	0.189	52	0.000	0.897	52	0.000 **
PANSS total	0.088	52	0.200 *	0.953	52	0.038 *
PQ-LES-Q total	0.107	52	0.194	0.952	52	0.036 *

This is a lower bound of the true significance. a. Lilliefors Significance Correction. * *p* < 0.05, ** *p* < 0.001.

**Table 2:** Demographic, clinical, and psychosocial characteristics of adolescents with schizophrenia and healthy controls.

Sample Characteristics	PAT-SCZ		HC		Chi Square Test (χ^2^) or Mann–Whitney U Test (PAT-SCZ vs. HC)	*p* Value
	N = 52	%	N = 51	%		
Gender						
males	23	44.2	23	45.1	χ^2^ = 0.008	0.93
females	29	55.8	28	54.9		
Family history of psychiatric disorders						
no	27	51.9	-	-	-	-
yes	25	48.1	-	-	-	-
Family relationships						
adequate relationships	31	59.6	-	-	-	-
dysfunctional relationships	21	40.4	-	-	-	-
Age (years): M (SD)	15.21 (SD = 1.27)		15.54 (SD = 1.22)		U = 1109.5, Z = −1.48	0.13
PQ-LES-Q score: M (SD)	56.78 (SD = 10.54)		72.88 (SD = 3.79)		U = 172.5, Z = −7.65	<0.0001 **
RMET score: M (SD)	18.23 (SD = 7.42)		29.19 (SD = 4.34)		U = 274.5, Z = −6.95	<0.0001 **
EQ-C sub-score: M (SD)	9.46 (SD = 4.11)		18.52 (SD = 2.67)		U = 97.5, Z = −8.12	<0.0001 **
EQ-AF sub-score: M (SD)	9.55 (SD = 3.52)		16.64 (SD = 3.78)		U = 239, Z = −7.19	<0.0001 **
EQ-SS sub-score: M (SD)	6.92 (SD = 2.19)		11.82 (SD = 1.88)		U = 88.5, Z = −8.21	<0.0001 **
EQ total score: M (SD)	25.94 (SD = 8.84)		47.00 (SD = 6.93)		U = 79, Z = −8.23	<0.0001 **
PANSS-P sub-score: M (SD)	7.57 (SD = 0.77)		-		-	-
PANSS-N sub-score: M (SD)	10.32 (SD = 2.69)		-		-	-
PANSS-G sub-score: M (SD)	20.13 (SD = 3.39)		-		-	-
PANSS total score: M (SD)	38.09 (SD = 5.58)		-		-	-
IQ score: M (SD)	97.04 (SD = 12.42)		101.27 (SD = 9.49)		U = 1094.5, Z = −1.53	0.12

** *p* < 0.001.

**Table 3:** Comparison of quality of life, social cognition, empathy, and psychopathology scores between normal metabolizers and reduced-function metabolizers.

Psychiatric Assessment Scales	PAT-SCZ-NM (N = 19)	PAT-SCZ-RM (N = 33)	Mann–Whitney U Test (PAT-SCZ-NM vs. PAT-SCZ-RM)	*p* Value
PQ-LES-Q score: M (SD)	67.47 (SD = 6.23)	50.63 (SD = 6.96)	U = 25, Z = −5.49	<0.0001 **
RMET score: M (SD)	23.94 (SD = 4.01)	14.94 (SD = 6.95)	U = 99.5, Z = −4.08	<0.0001 **
EQ-C sub-score: M (SD)	11.15 (SD = 2.16)	8.48 (SD = 4.65)	U = 176.5, Z = −2.62	0.009 *
EQ-AF sub-score: M (SD)	11.73 (SD = 3.67)	8.30 (SD = 2.78)	U = 123.5, Z = −3.65	<0.0001 **
EQ-SS sub-score: M (SD)	8.10 (SD = 0.99)	6.24 (SD = 2.41)	U = 146.5, Z = −3.21	0.001 *
EQ total score: M (SD)	31.00 (SD = 5.65)	23.03 (SD = 9.09)	U = 126, Z = −3.56	<0.0001 **
PANSS-P sub-score: M (SD)	7.47 (SD = 0.77)	7.63 (SD = 0.78)	U = 274.5, Z = −0.84	0.39
PANSS-N sub-score: M (SD)	8.42 (SD = 1.34)	11.42 (SD = 2.68)	U = 97.5, Z = −4.14	<0.0001 **
PANSS-G sub-score: M (SD)	17.63 (SD = 1.34)	21.57 (SD = 3.39)	U = 80, Z = −4.47	<0.0001 **
PANSS total score: M (SD)	33.68 (SD = 3.11)	40.63 (SD = 5.10)	U = 67, Z = −4.69	<0.0001 **
IQ score: M (SD)	104.15 (SD = 9.02)	92.93 (SD = 12.35)	U = 141, Z = −3.28	0.001 *

* *p* < 0.05, ** *p* < 0.01.

**Table 4:** Model 1—standard and bootstrapped linear regression estimates for predictors of PQ-LES-Q total scores.

Predictors	B	SE	β	t	*p*	Bootstrap 95% CI	VIF
RMET total scores	0.52	0.12	0.36	4.29	<0.001	[0.28, 0.73]	2.30
Metabolizer status	−7.85	1.58	−0.36	−4.97	<0.001	[−11.46, −4.21]	1.71
PANSS total scores	−0.50	0.17	−0.26	−2.96	0.005	[−0.82, −0.04]	2.54
Psychosocial context	−2.97	1.31	−0.14	−2.26	0.028	[−5.67, −0.03]	1.23

RMET: Reading the Mind in the Eyes Test; PANSS: Positive and Negative Syndrome Scale; Β = standardized coefficient; BCa 95% CI = Bias-corrected accelerated confidence interval based on 1000 bootstrap samples.

**Table 5:** Model 2—standard and bootstrapped linear regression estimates for predictors of PQ-LES-Q total scores.

Predictors	B	Std. Error	β	*p*	Bootstrap 95% CI	VIF
RMET total scores	0.49	0.12	0.35	<0.001 ***	[0.21, 0.75]	2.37
Metabolizer status	−7.40	1.60	−0.34	<0.001 ***	[−11.32, −3.57]	1.78
Psychosocial Context	−3.41	1.33	−0.16	0.013 *	[−5.93, −0.43]	1.26
PANSS (N) score	−0.79	0.30	−0.20	0.012 *	[−1.34, −0.07]	1.91
PANSS (P) score	0.68	0.80	0.05	0.402	[−1.10, 2.43]	1.12
PANSS (G) score	−0.44	0.24	−0.14	0.079	[−1.03, 0.20]	2.00

RMET: Reading the Mind in the Eyes Test; PANSS (N): Positive and Negative Syndrome Scale—Negative symptoms; PANSS (P): Positive and Negative Syndrome Scale—Positive symptoms; PANSS (G): Positive and Negative Syndrome Scale—General psychopathology; Β = standardized coefficient; BCa 95% CI = bias-corrected accelerated confidence interval based on 1000 bootstrap samples. * *p* < 0.05, *** *p* < 0.001.

**Table 6:** Comparative multivariable regression models for quality of life (PQ-LES-Q).

Predictor	Model 1 (Global)		Model 2 (Granular)	
	Std. β	Bootstrap 95% CI	Std. β	Bootstrap 95% CI
(Constant)	—	[54.58, 85.53] ***	—	[46.62, 81.42] ***
RMET total score	0.363	[0.28, 0.73] *	0.346	[0.21, 0.75] *
Metabolizer status	−0.362	[−11.46, −4.21] *	−0.341	[−11.32, −3.57] *
Psychosocial context	−0.139	[−5.67, −0.03] *	−0.160	[−5.93, −0.43] *
PANSS Total	−0.263	[−0.82, −0.04] **	—	—
PANSS Negative (N)	—	—	−0.201	[−1.34, −0.07] *
PANSS Positive (P)	—	—	0.050	[−1.10, 2.43]
PANSS General (G)	—	—	−0.141	[−1.03, 0.20]
				
Adjusted R^2^	0.842		0.843	
F-statistic	68.83 ***		46.78 ***	

* *p* < 0.05, ** *p* < 0.01, *** *p* < 0.001. Bootstrap results based on 1000 resamples. CI = bias-corrected accelerated confidence interval. Standardized Beta (β) values are reported for effect size comparison.

## Data Availability

Participants in this study did not provide written informed consent for the public release of their data. Given the sensitive nature of the research, supporting data cannot be provided.
